# The face of Dental Sleep Medicine in the 21st century

**DOI:** 10.1111/joor.13075

**Published:** 2020-08-29

**Authors:** Frank Lobbezoo, Gilles J. Lavigne, Takafumi Kato, Fernanda R. de Almeida, Ghizlane Aarab

**Affiliations:** ^1^ Department of Orofacial Pain and Dysfunction Academic Centre for Dentistry Amsterdam (ACTA) University of Amsterdam and Vrije Universiteit Amsterdam Amsterdam The Netherlands; ^2^ Faculty of Dental Medicine Centre d'étude du sommeil Université de Montréal and Hôpital du Sacré Coeur Montréal QC Canada; ^3^ Department of Oral Physiology Sleep Medicine Center Osaka University Hospital Osaka University Graduate School of Dentistry Osaka Japan; ^4^ Department of Oral Health Sciences Faculty of Dentistry University of British Columbia Vancouver BC Canada

**Keywords:** dental sleep medicine, obstructive sleep apnoea, oro‐facial pain, sleep, sleep bruxism

## Abstract

It becomes increasingly clear that some sleep disorders have important diagnostic and/or management links to the dental domain, hence the emergence of the discipline ‘Dental Sleep Medicine’. In this review, the following topics are discussed: 1. the reciprocal associations between oro‐facial pain and sleep; 2. the associations between sleep bruxism and other sleep‐related disorders; 3. the role of the dentist in the assessment and management of sleep bruxism; and 4. the dental management of obstructive sleep apnoea. From these topics' descriptions, it becomes clear that the role of the dentist in the recognition and management of sleep‐related oro‐facial pain, sleep bruxism and obstructive sleep apnoea is large and important. Since many dental sleep disorders can have severe consequences for the individual's general health and well‐being, it is imperative that dentists are not only willing to take on that role, but are also able to do so. This requires more attention for Dental Sleep Medicine in the dental curricula worldwide, as well as better postgraduate training of dentists who are interested in specialising in this intriguing domain. This review contributes to increasing the dental researcher's, teacher's and care professional's insight into the discipline ‘Dental Sleep Medicine’ as it has taken shape in the 21st century, to the benefit of all patients suffering from dental sleep disorders.

## 1 INTRODUCTION

We spend a significant proportion of our lives sleeping. Unfortunately, some of us suffer from sleep disorders, which can affect quality of life considerably. While most sleep disorders should be diagnosed and treated by medical doctors, it becomes increasingly clear that some disorders have important diagnostic and/or management links to the dental domain, hence the emergence of the discipline ‘Dental Sleep Medicine’ in dentistry. Dental Sleep Medicine is concerned with the study of the oral and maxillofacial causes and consequences of sleep‐related problems.^1^ Already more than twenty years ago, Prof. Lavigne and coworkers published a comprehensive review, entitled ‘Sleep disorders and the dental patient’, in which several common sleep disorders of interest to dentists were described, namely sleep‐related oro‐facial pain, oro‐facial movement disorders, breathing disorders, oral moistening disorders and gastro‐oesophageal reflux, all of which require the attention of dentists.^2^ In the present review, which is based on a symposium entitled ‘Wake‐up call: Dental Sleep Medicine is here to stay!’, held on June 20, 2019, during the IADR/AADR/CADR General Session & Exhibition, Vancouver, BC, Canada, the reciprocal associations between oro‐facial pain and sleep will be discussed first. Subsequently, the associations between sleep bruxism and other sleep‐related disorders will be discussed. Thereafter, the role of the dentist in the assessment and management of sleep bruxism will be explained, followed by an overview of the dental management of obstructive sleep apnoea, notably oral appliances. This article's ultimate goal is to increase the dental researcher's, teacher's and care professional's insight into the discipline ‘Dental Sleep Medicine’ as it has taken shape in the 21st century, to the benefit of all patients suffering from dental sleep disorders.

## 2 ORO‐FACIAL PAIN AND SLEEP

As to better understand the reciprocal associations between oro‐facial pain and sleep, a basic understanding of sleep itself is mandatory. Hence, below, the phenomenon ‘sleep’ will be briefly introduced first, followed by a description of the current insights into (oro‐facial) pain and its interaction with sleep.

### 2.1 Sleep

Sleep is a natural process that enables us to recover from our energy expenditure during wakefulness. It also contributes to mood maintenance, immune system recovery, brain and muscle regeneration, and memory consolidation. Sleep follows an approximately 24‐hour cycle under light and environmental cues. The sleep ‘centre’ is localised in the brainstem, with interplay from the suprachiasmatic nucleus and the thalamus. During sleep, the cortex is relatively quiet, except during specific phases of arousal that naturally occur every 20‐40 seconds. These arousals are cyclic physiological phenomena that occur to preserve homoeostasis and survival. Arousals are characterised by 3‐10 seconds increases in, amongst others: heart rate; autonomic nervous system, brain and muscle activities; and body temperature. Their main function is to preserve sleep stability or to trigger a full awakening that can be associated with a fight or flight survival reaction.^3^

### 2.2 Pain and sleep

Adult humans need to sleep between 7 and 9 hours to feel rested. Lack of sleep can be associated with bad sleeping habits or health issues (eg anxiety, depression, persistent pain, cancer). Sleep is not an anaesthesia or a coma, but rather an active state that filters external inputs. Pain during sleep, like any sensory input such as sound, will trigger arousals and, when needed, full awakenings to assure body protection.^4^ Interestingly, placebo analgesia also remains active during sleep as revealed by recent studies.^5^, ^6^ This all illustrates that pain influences sleep.

Reciprocally, sleep also influences pain. For example, it is recognised that 3‐4 nights of experimental sleep deprivation may trigger mood alteration and can initiate diffuse pain complaints in healthy subjects.^7^, ^8^ More recent studies confirmed that some dysfunctional processing in nociception can result from sleep deprivation, which will alter the activity of brain structures that are related to pain processing or reward modulation, for example enhanced activity at the cortex and reduced activity at the striatum, insula and nucleus accumbens.^9^, ^10^

### 2.3 Oro‐facial pain and sleep

Unrefreshing sleep is reported in many patients suffering from widespread musculoskeletal pains.^11^ About half of the oro‐facial pain patients, including patients with temporomandibular (TM) pain, report poor sleep quality, which suggests that oro‐facial pain negatively influences sleep quality.^12^ Reciprocally, a recent major study, the Orofacial Pain Prospective Evaluation and Risk Assessment (OPPERA) study, revealed that sleep quality is changed months before the novel onset of TM pain, which suggests that impaired sleep may also yield oro‐facial pain.^13^ In addition, such pre‐diagnostic changes in the trajectory of sleep quality support the fact that many patients with TM pain also present sleep disorders, such as insomnia, respiratory effort‐related arousal or sleep.^14^, ^15^, ^16^ Furthermore, TM pain patients also tend to present more mood alteration, such as depression symptomatology, or sleep and/or awake bruxism as compared to healthy controls.^17^, ^18^, ^19^

### 2.4 Conclusion

Form the above, it can be gathered that sleep and oro‐facial pain may indeed be associated in a bidirectional manner. This reciprocal association seems to be influenced by multiple biological and psychosocial factors, although the exact underlying mechanisms are still unknown. Dentists should be aware of this complex interplay, and, importantly, they should be able to deal with the related diagnostic and management issues. Suggested strategies for the management of the oro‐facial pain and sleep interaction by dentists are shown in Table 1. Even though the level of evidence is still rather ‘lean’ for the suggested management approaches, applying them in everyday dental practice will promote the professional collaboration between dentists and medical doctors specialising in sleep medicine.

**TABLE 1:** Suggested strategies for the management of the oro‐facial pain and sleep interaction by dentists^3^, ^4^, ^11^, ^12^

Assess whether comorbidities like bad life habits, mood alteration, bruxism or adverse oral habits are contributing to the oro‐facial pain condition
Suggest improvements in sleep hygiene, relaxation therapy, yoga, etc
Refer to psychologist or physical therapist when indicated
Confirm the role of other sleep disorders by a sleep physician who may conduct sleep testing/recording. With the exception of sleep bruxism without sleep comorbidities, the diagnosis and management of sleep disorders, such as insomnia, apnoea, periodic limb movement and REM behaviour disorders, are done by sleep physicians
Consider, after diagnosis of sleep disorders, an occlusal splint if no obstructive sleep apnoea is present, or a mandibular advancement device if headache and/or sleep‐breathing issues are present
Prescribe non‐steroidal analgesics and muscle relaxants as a management option for the short term. Avoid benzodiazepines or opioids if sleep‐disordered breathing, RERA or apnoea are suspected
Physicians may prescribe sleeping pills (eg zolpidem or off‐label trazodone as a sleeping aid), or other more powerful medication (eg duloxetine, amitriptyline, nortriptyline or pregabalin) to improve the deleterious pain and sleep interaction. Avoid pregabalin in combination with opioids because of an increased risk of breathing problems

## 3 **SLEEP BRUXISM AND OTHER SLEEP‐RELATED DISORDERS** 1

Sleep bruxism (SB) is a dental sleep disorder with which dentists are confronted regularly in their everyday practices. SB can be comorbid to many sleep‐related disorders.^20^, ^21^, ^22^ Insight into the underlying mechanism of these relationships may enable distinguishing between the primary and secondary forms of SB. In the absence of an underlying medical aetiology, SB is considered primary, or idiopathic, whereas secondary SB is associated with a medical condition. The distinction between these two forms is important, because their management may be distinct. In cases where the primary form of SB has harmful consequences, management of SB is often necessary. However, when SB is a comorbid condition of other sleep‐related disorders, management of the associated medical conditions by an expert physician should be the first focus. It is to be expected that the management of the sleep‐related disorder may prevent or reduce SB and its consequences on dental and general health.

### 3.1 Sleep bruxism and obstructive sleep apnoea

**FIGURE 1:**
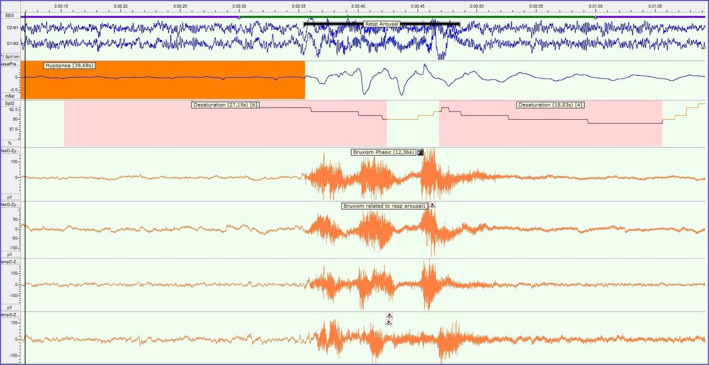
Example of a 1‐min page of a polysomnographic recording, displaying, from top to bottom, two electro‐encephalographic (EEG) leads, an airflow channel, an oxygen saturation channel and four electromyographic (EMG) leads of the masseter and anterior temporalis muscles from both sides. Clearly visible are the following events: arousal in both EEG leads, hypopnea event in the airflow channel, oxygen desaturation in the oxygen saturation channel and rhythmic masticatory muscle activity in the EMG leads. In this example, the onset of the breathing event precedes the onset of the sleep bruxism (ie rhythmic masticatory muscle activity; RMMA) event

The prevalence of SB in adult obstructive sleep apnoea (OSA; for more details, see ‘Obstructive sleep apnoea: the dentist's role’) patients is much higher than in controls, namely 26% based on self‐report,^23^ and ranges from 34% to 65% based on polysomnographic (PSG) data.^24^, ^25^ This may suggest that both phenomena are associated with each other. Recently, four hypothetical scenarios for a temporal relationship between SB and OSA were proposed^22^: (a) The two phenomena are unrelated; (b) the onset of the OSA event precedes the onset of the SB event, within a limited time span, with SB having a potential OSA‐protective role (see Figure 1); (c) the onset of the SB event precedes the onset of the OSA event, within a limited time span, with SB having an OSA‐inducing effect; and (d) the onset of the OSA and SB event occurs at the same moment. The authors concluded that the findings on the SB‐OSA temporal relationship are still inconclusive. In addition, the relative predominance of one specific sequence of events varies at the individual level.

Apart from the four hypothetical scenarios, age may also be critical for the association.^26^, ^27^ On the one hand, the prevalence of SB self‐reports shows a natural course of reduction over the life time span, while on the other hand, the prevalence of OSA increases with increasing age, which suggests that the two phenomena may be concomitant and are physiologically unrelated. As proposed previously and reiterated in the hypothesis paper, SB activity may also have a protective effect against OSA by protruding the mandible and subsequently improving airway patency.^22^, ^28^ Clearly, to deepen our insight into the putative association between SB and OSA, experimental and clinical studies for exploring the underlying pathophysiological mechanisms, large data bank studies for the assessment of risk factors, and longitudinal clinical studies for causality are required.^29^

### 3.2 Sleep bruxism and restless leg syndrome/ periodic leg movements in sleep

The four cardinal diagnostic features of restless leg syndrome (RLS) include the following: (a) an urge to move the limbs, which is usually associated with paraesthesias or dysaesthesias; (b) symptoms that start or become worse with rest; (c) at least partial relief of symptoms with physical activity; and (d) worsening of symptoms in the evening or at night.^30^ In addition, the patient must note a symptom of concern, distress, sleep disturbance or some impairments related to the sensations. Frequently, RLS also has a primary motor symptom that is characterised by the occurrence of periodic leg movements in sleep (PLMS). The resulting brief arousals can contribute significantly to disturbed sleep. PLMS occur in approximately 80%‐90% of patients who have RLS.^30^

Van der Zaag et al^21^ reported that the PLMS index (ie the number of PLMS events per hour of sleep), based on PSG recordings, was significantly higher amongst Dutch SB patients compared to healthy controls. Within the group of SB patients, the combined SB/PLMS index was higher than the isolated PLMS index and the isolated SB index. Further, the combined SB/PLMS index with electro‐encephalographic (EEG) arousals was significantly higher than the combined SB/PLMS index without EEG arousal in SB patients. Therefore, the authors concluded that SB and PLMS probably have a common underlying neurophysiologic mechanism. Also, the pharmacologic management of both disorders shares some similarities. There have been reports suggesting the effectiveness of dopaminergic agonists on the treatment of SB and of PLMS, which may support a common underlying neuropharmacologic mechanism as well, although the effectiveness of such medication is higher in PLMS than in SB.^31^, ^32^ In addition, age may also be critical for the association between SB and RLS/PLMS: while the prevalence of SB is decreasing with increasing age, that of RLS/PLMS increases with ageing,^33^ which suggests that the two phenomena may be concomitant and are physiologically unrelated. Future studies with larger sample sizes and PSG validation are required to draw conclusions on SB as a comorbid condition of RLS/PLMS.

### 3.3 Sleep bruxism and insomnia

Insomnia is defined as a sleep complaint that occurs at least three times per week for at least 3 months and is associated with daytime impairment.^34^ The prevalence of insomnia in the general population ranges from 4% to 48%, depending on the definition of insomnia used and the methods used for determining the condition.^35^ According to recent studies, bruxers have more difficulties with initiating sleep, complain more about a disturbed sleep and tend to report more excessive daytime sleepiness than controls.^36^, ^37^ Further, some SB patients also report problems with maintenance of sleep.^37^ In another study, a positive association between SB and insomnia symptoms was observed in the general population in Brazil, using a sample of 1042 individuals who answered questionnaires and underwent PSG for SB diagnosis.^38^ Despite these consistent findings, more research is needed to definitely confirm such an association, to assess the effect of ageing on the association and to clarify its relevance in comorbid SB management.

### 3.4 Sleep bruxism and REM sleep behaviour disorder

REM sleep behaviour disorder (RBD) is a parasomnia characterised by the presence of abnormal motor behaviours during REM sleep.^34^ RBD can be associated with neurodegenerative disorders like Parkinson disease (PD) and dementia. In fact, RBD precedes neurodegenerative disorders in over 30% of cases at 5 years and in 90% of cases at 14 years after the establishment of the RBD diagnosis.^39^ Two studies have reported on the association between SB and RBD with concomitant PD.^20^, ^40^ In Finnish patients with PD, the prevalence of SB was lower than that of the general population (5% vs 8%, respectively) based on self‐report.^40^ On the other hand, in a Canadian PSG study, it was found that both in patients with RBD only and in patients with concomitant RBD and PD, the rhythmic masticatory muscle activity (RMMA) index was significantly higher than in a control group.^20^ In addition, in patients with RBD, the oromandibular myoclonus index was significantly higher than in the control group. The authors therefore suggested that in the presence of a tooth tapping complaint and a high frequency of RMMA during REM sleep, RBD may be suspected and further neurological assessment is recommended.^20^ Future longitudinal studies monitoring RBD patient groups on the occurrence of SB and PD can further deepen our understanding of these conditions' correlations and may facilitate identifying the early presentation of PD or other neurodegenerative disorders.

### 3.5 Sleep bruxism and sleep‐related gastro‐oesophageal reflux disease

Sleep‐related gastro‐oesophageal reflux disease (GERD) is characterised by regurgitation of stomach contents into the oesophagus during sleep. Shortness of breath and/or heartburn can result, but occasionally the disorder is asymptomatic (‘silent reflux’). Experimental and clinical evidence from studies done in Japan supports that SB and sleep‐related GERD can be associated, at least in some cases.^41^, ^42^ In an experimental study, in which polysomnography, audio‐video recording and oesophageal pH monitoring in 12 healthy adult males were performed, it was shown that intra‐oesophageal acidification induces SB.^42^ In another small sample size (n = 10) clinical study, the authors concluded that nocturnal bruxism may be secondary to nocturnal gastro‐oesophageal reflux, occurring via sleep arousal and occurring with swallowing.^41^ In short, the above‐described evidence suggests that SB is associated with GERD.

### 3.6 Conclusion

Although the evidence on the relation between SB and other sleep‐related disorders is scarce and consequently limits our interpretation, it is suggested that SB and other sleep‐related disorders are in most cases associated with sleep arousals. The likelihood of arousals, which can be part of the physiology related to a certain sleep‐related disorder, potentially influences the probability of SB occurrence. In patients with concomitant SB and other sleep‐related disorders, SB may thus be considered as a secondary form of SB. Management of secondary SB with negative health consequences should therefore be done in collaboration with expert physicians.

## 4 **SLEEP BRUXISM: THE DENTIST**'**S ROLE**

The management of the primary and secondary forms of SB is one of the most challenging and debated topics in dentistry and sleep medicine. This section will first focus on the management of primary SB, followed by a description of the challenges of assessing and managing secondary forms of SB.

### 4.1 Primary sleep bruxism

Over the years, several management strategies have been proposed for clinically controlling primary SB activity.^43^ As dental strategies, occlusal splints are often used for protecting the teeth against mechanical damage from SB. Mandibular advancement devices for opening the upper airway have recently been proposed for the management of SB as well when sleep‐related breathing disorders are suspected or confirmed. Pharmacological strategies are still not ready for application in patient care yet, although a few drugs, such as clonidine, L‐dopa, clonazepam and proton‐pump inhibitors, have been shown to reduce SB. Amongst the behavioural approaches, biofeedback systems have been on market for a while. However, due to limitations of the methodologies (eg small study samples, short‐term follow‐up only) and the small number of studies, we still cannot determine the definitive management strategy for reducing SB on the basis of solid scientific evidence.^44^, ^45^, ^46^ Interestingly, in many clinical studies, differences in the individual treatment responses can be found, even though pooled data did not show statistically significant effects. This suggests that responders and non‐responders can be expected for a given management strategy. Nonetheless, unlike OSA for which different phenotypes have been described in relation to treatment outcomes,^47^ we have not yet identified or classified the pathophysiological or clinical phenotypes of SB.

### 4.2 Secondary sleep bruxism

As has been discussed in the previous section, SB has been reported to concomitantly occur with other sleep‐related disorders.^48^ Currently, it is not known whether a concomitant occurrence of SB with other sleep disorders represents secondary influences of sleep fragmentation, common pathophysiological mechanisms or a coexistence of independent entities. However, comorbidity with other sleep disorders can represent clinical phenotypes of SB, because clinical signs and symptoms of SB can be modified or shared by other sleep disorders. Subjective report of tooth grinding can be over‐estimated in patients with TM disorders (TMD), stress, and/or anxiety, the conditions that put one at risk of poor sleep quality: high correlations with self‐reported SB were found to diminish when SB was assessed more objectively.^49^, ^50^, ^51^ Tooth wear can be severe not only in patients with SB but also in patients with OSA, TMD and/or gastro‐oesophageal reflux.^52^ Morning headache and jaw symptoms can often be associated with primary snoring, OSA and primary insomnias.^53^, ^54^, ^55^ Finally, we should recognise that most risk factors for SB (eg stress, smoking, caffeine, alcohol, chronic oro‐facial pain)^56^ are known as risk factors for sleep disturbance and sleep disorders in sleep medicine as well.^34^

Comorbidity between SB and other sleep disorders can be a confounding factor when clinical assessment of SB is confirmed by an objective or physiological assessment. In dentistry, portable EMG recordings (type III or IV level examination in sleep medicine) have preferably been used to detect and quantify ‘sleep‐related’ masticatory EMG activity.^57^ As SB patients frequently exhibit RMMA during sleep, RMMA is used as a physiological marker for diagnosing SB. However, sleep‐related masticatory EMG activity can also be found in patients with frequent occurrence of sleep arousals (eg OSA) and abnormal motor events (eg PLMS, RBD).^48^ Polysomnography (type I and II level examination) can provide us with information on pathophysiological details of SB and comorbid sleep disorders. PSG diagnosis of SB has been validated using the clinical diagnosis of SB as the reference standard.^34^, ^58^ However, since PSG is associated with high cost and requires manpower, it cannot be a routine examination for dental patients.

### 4.3 Conclusion

Dentists will frequently be encountered with patients with secondary SB and comorbid sleep disorders. Screening for sleep complaints and symptoms is one way to improve chairside assessment: it will give an impression of clinical phenotypes and will be helpful to understand the patients' oro‐facial symptoms and complaints related to personalised management (Table 2).^48^, ^59^ If necessary, SB patients can be referred to sleep specialists for a detailed examination with PSG or even for treatment of comorbid sleep disorders.

**TABLE 2:** Examples of items to assess by dentists when screening for sleep complaints and symptoms^48^, ^59^

1: Life style – Circadian rhythm, regularity of sleep/wake rhythm – Stress, related events – Other habits: smoking, alcohol, caffeine, opioid use
2: Sleep‐related complaints – Sleepiness, fatigue, unrefreshed sleep – Difficulty falling sleep, frequent awakening
3: Signs/symptoms of sleep disorders – Reports of abnormal events:
(a) Related to OSA: loud snoring and breathing stops
(b) Related to NREM and REM parasomnias and sleep‐related movement disorders: abnormal behaviours and movements, sound/noise (eg talking)
– Physical observations and examination:
(a)Related to OSA: obesity, neck circumstance, craniofacial morphology (micrognathia, macroglossia, tonsillar/adenoid, Mallampati classification)
(b) Related to RLS: an urge to move the legs with uncomfortable/unpleasant sensation in the evening or at bedtime
4: Systemic diseases and medication affecting sleep complaints/somnolence – Systemic diseases: psychiatric (eg depression), neurological (eg Parkinson's disease, epilepsy), cardiovascular (eg hypertension), endocrine (eg diabetes), respiratory (eg chronic obstructive pulmonary disease), chronic pain, gastro‐oesophageal reflux – Medication: antidepressants (eg SSRIs, TCAs, MAO inhibitors), anxiolytics (eg benzodiazepines), antipsychotics, antiepileptics, histamine antagonists, cardiovascular drugs (eg beta‐adrenergic blockers)

## 5 **OBSTRUCTIVE SLEEP APNOEA: THE DENTIST**'**S ROLE**

OSA is a key condition for dentists specialising in Dental Sleep Medicine. Below, OSA will be introduced in more detail, after which the role of dentists with various professional backgrounds in OSA is described from a North American point of view.

### 5.1 Obstructive sleep apnoea

OSA is characterised by repetitive complete or partial closures of the upper airway, and is associated with snoring, daytime sleepiness, poor sleep quality and various cardio‐metabolic health problems. With the upper airway collapse, there is a cascade of events, such as (a) decrease in oxygen and an increase in carbon dioxide, followed by an increase in the sympathetic activation and a decrease in vagal tone, which are responsible for an increase in blood pressure, heart rate and the renin‐angiotensin‐aldosterone system activity; (b) an arousal, which is a brief awaking from 3‐10 seconds that leads to sleep fragmentation; (c) fluctuation in the intra‐thoracic pressure, leading to left ventricular remodelling; and (d) increase in oxidative stress reading to endothelial activation and coronary artery disease.^60^

OSA is a common disease, and its prevalence increases with age and weight gain. According to a comprehensive epidemiological study,^61^ OSA prevalence between the ages of 50 and 70 years old is 36% and 61% for overweigh and obese men, respectively. The prevalence in women also follows increase in weigh, but is about a third of the prevalence found in men. From this, the high burden of OSA in the adult population can be gathered, as well as the necessity of treatment for a large percentage of the population.

### 5.2 Oral appliances

Dental Sleep Medicine has taken its largest step with the introduction of oral appliances (OAs) as a treatment for OSA in the late 1980s, but it was not until the late 1990s and early 2000 that OAs had enough scientific evidence to be fully accepted by the medical community as an effective alternative treatment for patients who could not tolerate continuous positive airway pressure (CPAP).^62^ After this step, there have been many studies showing comparable effectiveness between OA and CPAP, where, despite CPAP being more effective reducing the apnoea‐hypopnea index (AHI), in general a much lower acceptance and adherence of CPAP was shown.^63^ Importantly, studies comparing cardio‐metabolic and subjective outcomes have shown no differences between the two treatments.^64^

### 5.3 Dentist's role in obstructive sleep apnoea

As since the mid 1990s dentists started seeing more and more patients with sleep apnoea, it was obvious that dentistry had a larger role other than only providing OA treatment. There are various aspects where dentistry can contribute to the field of sleep medicine, especially in OSA. An overview of the focus and role of various dental specialists in the assessment and management of OSA is provided in Table 3.

**TABLE 3:** Focus and role of various dental specialists in the assessment and management of obstructive sleep apnoea (order as they appear in the text)

Specialty	Focus	Role
Orthodontist	Growth and development	Recognising, screening, treatment, referral
Pedodontist	Syndromic children	Recognising, screening, treatment
Radiologist	Anatomical factors	Upper airway imaging
Anaesthesiologist	Risk management	Recognition, referral
Periodontist	Similar pathways to disease	Impact of periodontal treatment on OSA
Gerodontologist	Higher incidence in elderly	Screening, assessment, treatment
Oral and Maxillofacial Surgeon	Maxilla‐mandibular advancement	Treatment, follow‐up
General Dentist	Oral Appliance therapy	Recognising, screening, treatment, follow‐up
Prosthodontist	Denture impact on upper airway patency	Assessment, treatment
Implantologist	Oral appliance in edentulous individuals	Assessment, treatment
TMD/Oro‐facial Pain Specialist	Side effects of oral appliance	Assessment, follow‐up

As a starting point, craniofacial development has for long been a field of research in orthodontics and paediatric dentistry. It is known that many syndromes that affect growth and development have an underdevelopment of the maxilla and/or mandible in common, which is highly associated with OSA.^65^ Also, children with micrognathia or midface hypoplasia are at higher risk of OSA.^66^ This is an important area for further development, as for now, skeletal malocclusion has not been shown to be more prevalent in OSA children compared to children without sleep‐disordered breathing.^67^, ^68^

In the field of oral and maxillofacial radiology (OMFR), dentists commonly prescribe panoramic and cephalometric radiographs and are able to identify calcification of the carotid artery, which is a common condition amongst OSA patients.^69^ More recently, with the advance of 3D imaging, cone‐beam CT has been used to evaluate the craniofacial complex in three dimensions and to assess the upper airway.^70^, ^71^

Dental anaesthesiology is linked to OSA, because OSA patients are often prone to increased risk of complications during deeper sedation. Anaesthesiologist have an important role in screening of the patients, the early recognition of OSA, and caring for a patent upper airway during deeper sedation procedures.^72^

Patients with OSA present with oxidative stress and systemic inflammation.^73^, ^74^ Periodontal disease shares some of these mechanisms, and recent studies have shown a significant association with OSA.^75^, ^76^ However, further studies are necessary to understand the role of obesity as a confounding factor.^77^ Also, studies to the effect of treatment of OSA on periodontal disease and *vice versa* are required.

As individuals age, there are several health issues that occur, such as an increase in the incidence of OSA, periodontal disease, diabetes and teeth loss. It has been proposed that when individuals are young, if there is a complete loss of teeth, this can be a promoter of OSA, while in older people, the use of dentures during sleep could positively affect the severity of OSA, serving as a treatment for some, but not all individuals.^78^, ^79^ Gerodontologists thus have an important role in the assessment of these patients, and may help screening and even treating OSA in selected cases.

As part of the treatment spectrum of OSA,^80^ surgical maxilla‐mandibular advancement by an oral and maxillofacial surgeon has been shown to be one of the best strategies, with a 90% chance of a reduction of 50% or more in the AHI, albeit in selected cases only and with a considerable risk of complications.^81^

General dentists see their patients on a regular basis for dental cleaning and treatment. Dentist are able to understand the individual's symptoms such as sleepiness and often check on health status, thus making them well suited for the role of raising awareness and referring patients for OSA assessment.^82^ With a broad training and higher involvement with ongoing care, general dentists are able to provide OA treatment. OA therapy requires further sleep training. It is more complex than one may imagine at first glance, while highly rewarding when treatment success can be achieved.^83^

Consequences of the treatment with oral appliances are bite changes, with a mesialisation of the lower dentition and distalisation of the upper dentition.^84^, ^85^ Unfortunately, despite some attempts,^86^ the prevention of these side effects has not been possible and the management of the changes in the occlusion have to be continuous as we know the dental changes are small but will continue as long as patients continue OA treatment. Orthodontists,^87^ prosthodontists, implantologists and general dentists have to work together, and also with speech therapists, to better follow the patients and find new approaches to prevent and/or manage dental side effects. Specialist on the temporomandibular complex has found an increase of side effects in the beginning of treatment but not at the long term,^88^ and bite changes have been solely related to dental angulations.^89^ Longer follow‐up research is still required to understand possible predictors of side effects, possible decreased efficacy of OAs with age and management of adherence.^90^

### 5.4 Conclusion

Patient‐centred care is the future, where we, as health care providers, need to (a) better understand and respect the patients' specific needs and their specific clinical and physiological phenotype, their preferences, their psychological status, expectations and beliefs; (b) help them in their decision making, give them education and support, engage them to the treatment by proving health apps and access to treatment, discussion platforms and advocacy groups; (c) develop patient‐centred measures for research; and (d) have dentists involved in the care coordination, continuity and access to care. In short, dental clinicians and researchers have an important role caring for patients with OSA and developing new knowledge, together with many other specialties in the field of OSA.

## 6 CONCLUDING REMARKS

This article aimed to increase the dental researcher's, teacher's and care professional's knowledge on Dental Sleep Medicine, to the benefit of all patients suffering from dental sleep disorders. The paper can be seen as a next step towards an increased awareness of the importance of this relatively new dental discipline. Traditionally, Dental Sleep Medicine mainly deals with OSA. While the landmark publication of Lavigne et al^2^ tried to initiate the broadening of the domain with several other dental sleep disorders (see above), the paper attracted only limited attention. Likewise, the paper describing a new definition for Dental Sleep Medicine^1^ remained largely unnoticed as well, as evidenced by the low number of citations. As to increase awareness, the definition paper was republished in a specialised journal.^91^ In addition, two Letters to the Editor on the new definition of Dental Sleep Medicine were published in (dental) sleep journals.^92^, ^93^ Together with the present paper, it is the authors' ambition that awareness will finally descend that in the 21st century there is more to Dental Sleep Medicine is a broad dental discipline.

In this article, first the phenomenon ‘sleep’ was briefly introduced, after which the bidirectional associations between sleep and (oro‐facial) pain were described. Thereafter, two of the most prevalent dental sleep disorders, namely SB and OSA, were outlined. For SB, the condition's associations with other sleep‐related disorders were described, while the role of the dentist was sketched in the recognition and management of sleep‐related oro‐facial pain, SB, and OSA. Clearly, that role is large and important. Since many dental sleep disorders can have severe consequences for the individual's general health and well‐being, it is imperative that dentists are not only willing to take on that role, but are also able to do so. This requires more attention for Dental Sleep Medicine in the dental curricula worldwide, as well as better postgraduate training of dentists who are interested in specialising in this intriguing domain.^94^, ^95^ The above information also shows that there are still considerable gaps in our knowledge on dental sleep disorders, on how these disorders are causally linked to each other as well as to other sleep disorders, and on which biological and psychosocial factors are involved in these associations. As a consequence of all this, more research is needed, which will ultimately be to the benefit of our patients.

## CONFLICT OF INTEREST

The authors declare that they received no funding for this study. The have also stated explicitly that there are no conflicts of interest in connection with this article.

## AUTHOR CONTRIBUTIONS

All authors contributed substantially to the conception, drafting and critical revision of this work. All authors have approved the final version for publication and are fully accountable for all aspects of the work.

### Peer Review

The peer review history for this article is available at https://publons.com/publon/10.1111/joor.13075.

## Footnotes

^1^ This paragraph is partly based on the following book chapter: Aarab G, Balasubramaniam R, Maluly Filho M, Lavigne GJ. Sleep Bruxism as a co‐morbid condition of other sleep‐related disorders. In: Lavigne GJ, Cistulli PA, Smith MT (eds). Sleep Medicine for Dentists: An Evidence‐Based Overview, 2nd ed. Batavia, IL: Quintessence Publishing Co, Inc, 2020. With permission from the publisher.
